# The guidance of stem cell cardiomyogenic differentiation by bioartificial scaffolds mimicking myocardium structure and biomechanics

**DOI:** 10.1186/1878-5085-5-S1-A81

**Published:** 2014-02-11

**Authors:** Caterina Cristallini, Elisa Cibrario Rocchietti, Lisa Accomasso, Anna Folino, Clara Gallina, Luisa Muratori, Pasquale Pagliaro, Raffaella Rastaldo, Stefania Raimondo, Silvia Saviozzi, Andrea Sprio, Niccoletta Barbani, Claudia Giachino

**Affiliations:** 1 CNR-IMCB, UOS, Pisa, Italy; 2 Department of Clinical and Biological Sciences, University of Turin, Italy; 3 Department of Civil and Industrial Engineering, University of Pisa, Italy

## Scientific objectives

Despite enormous progresses in the treatment of coronary artery disease, it remains the most common cause of heart failure and the leading cause of death in the Western countries. New translational therapeutic approaches based on personalized and regenerative medicine explore cardiomyogenic differentiation of various types of stem cells by electrical stimulation, biochemical inducers, or cell co-culturing [1-3]. In this study we fabricated bioartificial constructs mimicking anisotropic structure and mechanical properties of the myocardium [4].

## Technological approaches

Constructs based on PHBHV and gelatin were prepared and characterized by physico-chemical, mechanical and degradation tests including FT-IR, DSC, DMA and HPLC analyses. Cell adhesion and proliferation on these constructs were evaluated at 1, 4, 8, 12, 15 days using CellTiter Blue® viability assay. Cell morphology and construct colonization was evaluated using the viable dye Calcein-AM, while cytoskeletal organization and focal adhesions was analyzed by immunofluorescence with phalloidin staining and TEM. Cardioinductivity was investigated through qPCR (gene expression) and immunofluorescence (protein expression) experiments.

## Results interpretation

**Figure 1:**
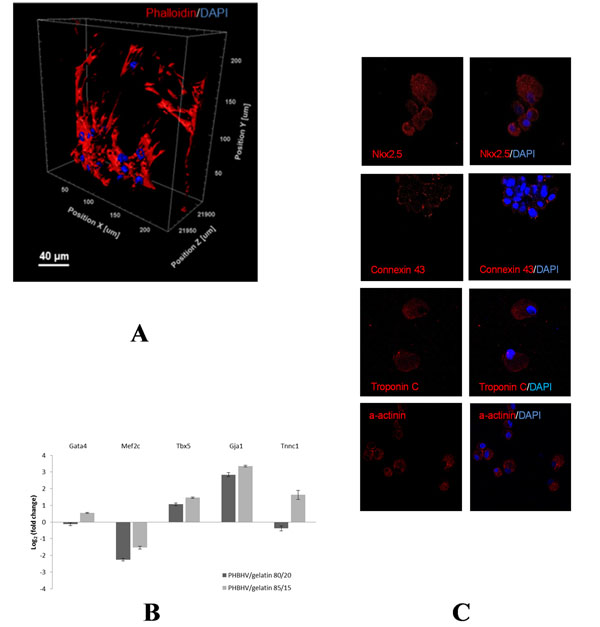
(A) Representative 3-D reconstruction of a Z-stack confocal image showing arrangement of NMCs on a PHBHV/gelatin scaffold after 15 days of culture. Actin filaments are stained in red (phalloidin) and nuclei are in blue (DAPI). Gene (B) and protein (C) expression analyses of cardiac differentiation markers.

Chemical structure and molecular interaction between material components induced specific properties to the substrate in terms of hydrophilicity degree, porosity, chemical compatibility and mechanical characteristics. Viability and proliferation assays demonstrated that these constructs permit mesenchymal stem cell (MSC) and cardiac resident non myocytic cell (NMC) adhesion and growth. Moreover, with confocal microscopy analysis we demonstrated that stem cells adopt a highly stretched and thin morphology and a distribution mimicking the 3-D cell alignment of myocardium (Fig. 1A). qPCR and protein analyses, performed after 15 days of culture on PHBHV/gelatin constructs, showed the ability of this structure to direct initial MSC and NMC lineage specification towards cardiomyogenesis in the absence of any external stimuli (Fig. 1B and 1C). Both MSCs and NMCs showed the expression of cardiac transcription factor GATA-4 that plays an essential role in early cardiac differentiation, and NMCs also acquired the expression of later cardiac transcription factors as Tbx5 and Nkx 2.5, which in turn led to the expression of functional and structural proteins such as Connexin 43, Troponin C and α-actinin.

## Outlook and expert recommendations

This work represents a new approach to induce both circulating and resident stem cells to differentiate toward cardiac cells in a 3-D structure, without using any additional stimuli. These constructs have the potential to serve as patches for cardiac regeneration.

Our results suggest that the two technology-driven components of the healthcare revolution, personalized medicine and regenerative medicine, offer the opportunity to address unmet medical needs.
